# Large-scale semantic indexing of Spanish biomedical literature using contrastive transfer learning

**DOI:** 10.1093/bioinformatics/btag794

**Published:** 2026-10-24

**Authors:** Ronghui You, Tianyang Huang, Ziye Wang, Yuxuan Liu, Hong Zhou, Shanfeng Zhu

**Affiliations:** School of Statistics and Data Science, LPMC, Nankai University, Tianjin 300071, China; Institute of Science and Technology for Brain-Inspired Intelligence and MOE Frontiers Center for Brain Science, Fudan University, Shanghai 200433, China; School of Mathematical Sciences and LPMC, Nankai University, Tianjin 300071, China; Shanghai Key Lab of Intelligent Information Processing and Shanghai Institute of Artificial Intelligence Algorithm, Fudan University, Shanghai 200433, China; Head Office, KnowledgeWorks Global Ltd, Glen Allen, VA 23059, United States; Institute of Science and Technology for Brain-Inspired Intelligence and MOE Frontiers Center for Brain Science, Fudan University, Shanghai 200433, China; Shanghai Key Lab of Intelligent Information Processing and Shanghai Institute of Artificial Intelligence Algorithm, Fudan University, Shanghai 200433, China; Key Laboratory of Computational Neuroscience and Brain-Inspired Intelligence, Fudan University, Ministry of Education, Shanghai 200433, China; Zhangjiang Fudan International Innovation Center, Shanghai 200433, China

## Abstract

**Motivation:**

The exponential growth of biomedical literature has made automatic indexing essential for advancing biomedical research. While automatic indexing has made strides in English biomedical literature, there has been limited research on non-English biomedical texts due to insufficient high-quality training data.

**Results:**

We propose BERTDeCS, a novel deep learning framework for automatically indexing Spanish biomedical literature using contrastive transfer learning. BERTDeCS utilizes a multilingual BERT (M-BERT) to generate multi-language representations and adapts M-BERT for Spanish biomedical literature domain through contrastive learning. Additionally, BERTDeCS enhances its semantic indexing capabilities on Spanish biomedical literature by leveraging enriched English annotated literature in MEDLINE through transfer learning. Experimental results on Spanish datasets demonstrate that BERTDeCS outperforms state-of-the-art indexing methods, achieving top performance in the MESINESP and MESINESP2 Tasks on medical semantic indexing in Spanish within the BioASQ challenge. Additional experiments on Portuguese provide preliminary evidence that BERTDeCS can transfer to a closely related language sharing substantial lexical and biomedical terminology with Spanish.

**Availability:**

The source code is available at https://github.com/yourh/BERTDeCS and at https://doi.org/10.5281/zenodo.23212468.

## 1 Introduction

As the volume of biomedical literature continues to surge, reaching millions of new publications annually (https://www.nlm.nih.gov/bsd/medline_pubmed_production_stats.html), health professionals encounter challenges accessing pertinent information on specific topics. Biomedical semantic indexing is crucial in information retrieval, knowledge extraction, and discovery from vast literature databases (Lu *et al*. 2009, Stokes *et al*. 2009, Zhu *et al*. 2009, Gu *et al*. 2013). However, manual indexing is a time-consuming, expensive, and labor-intensive process. Therefore, it is essential to develop highly accurate large-scale automatic semantic indexing models for biomedical literature.

Since the inception of the BioASQ (a challenge on large-scale biomedical semantic indexing and question answering) in 2013, there have been significant advancements in semantic indexing algorithms for English biomedical literature (Liu *et al*. 2017, Jin *et al*. 2018, Dai *et al*. 2020, You *et al*. 2021, Nentidis *et al*. 2022, 2021, Krithara *et al*. 2023). This enhancement in performance prompted the U.S. National Library of Medicine (NLM) to implement fully automated indexing for MEDLINE since 2022 (https://www.nlm.nih.gov/mesh/intro_indexing.html). In contrast, automated semantic indexing of non-English biomedical literature has not achieved the desired outcomes yet.

Here we focus on semantic indexing of Spanish biomedical literature. Spanish ranks as the second most widely spoken language by native speakers, with more than 477 million individuals. According to WHO statistics, Spain alone has over 180 000 active physicians, more than 247 000 nursing and midwifery professionals, and 55 000 pharmaceutical personnel (https://temu.bsc.es/mesinesp2/about-the-task). To facilitate healthcare professionals and researchers who use Spanish or Portuguese, BIREME (Latin American and Caribbean Center on Health Sciences Information) established a multilingual digital platform named VHL (https://bvsalud.org/en/) (Virtual Health Library) in 1998 (Linares and Larsen 2023). The VHL aims to bridge the gap between knowledge and health practice in Latin American and Caribbean countries by providing access to scientific and technical information in the health sector. It centralizes and provides comprehensive medical and health science resources in various languages, including English, Spanish, Portuguese, and other local languages.

To enhance the semantic indexing of literature available in the VHL, BIREME developed DeCS (Health Sciences Descriptors) (Pellizzon 2004, Campanatti-Ostiz and Andrade 2010), which is a multilingual (English, Spanish, Portuguese, and French) and structured vocabulary derived from MeSH (Medical Subject Headings) (Lipscomb 2000) of the NLM. In addition to MeSH, DeCS includes terminology in five unique specific areas: Science and Health, Homeopathy, Traditional, Complementary and Integrative Medicine, Public Health, and Sanitary Surveillance. To date, DeCS has a total of 77 qualifiers and 34 664 descriptors, of which 30 766 (88.75%) come from MeSH, and 3898 are exclusively DeCS’ (https://decs.bvsalud.org/en/about-decs). The enriched indexing system provided by DeCS improves the retrieval and accessibility of biomedical literature, facilitating knowledge dissemination and promoting collaboration among researchers, healthcare professionals, and other stakeholders. However, the large scale of DeCS presents a significant challenge due to the substantial human and material resources required for high-quality manual semantic annotation.

In this work, we propose a new deep learning-based model, namely BERTDeCS, to improve the performance of automated semantic annotation for Spanish biomedical literature. In recent years, several Spanish biomedical and clinical pre-trained encoders have been developed, such as roberta-base-biomedical-es (Carrino *et al*. 2021) and EriBERTa (de la Iglesia *et al*. 2023). These models provide useful domain-specific text representations for Spanish or Spanish–English biomedical text. However, the main challenge in Spanish DeCS indexing is not only the choice of encoder. Compared with English MeSH indexing in MEDLINE, Spanish DeCS indexing has substantially fewer manually indexed training instances. Therefore, BERTDeCS focuses on adapting multilingual representations and transferring large-scale English indexing knowledge to Spanish DeCS prediction. BERTDeCS leverages M-BERT (multilingual BERT (Devlin *et al*. 2019)) for processing Spanish literature and adopts a multi-label attention mechanism to capture the most relevant parts for each label. Given the close associations of semantic indexing of Spanish and English literature, we employ a transfer contrastive learning approach to address the fundamental challenges encountered in the automatic indexing of Spanish biomedical literature, including insufficient annotations and low annotation quality. Specifically, to make M-BERT more suitable for handling Spanish biomedical literature, BERTDeCS fine-tunes M-BERT using a bilingual-based contrastive learning approach. Additionally, considering the substantial overlap between MeSH and DeCS codes, we leverage the high-quality manual semantic annotations from the large-scale English biomedical literature in MEDLINE (>30 million citations) with a transfer learning strategy. We pre-train BERTDeCS on the large-scale English literature of MEDLINE and then fine-tune it on the Spanish literature of VHL. This transfer learning strategy allows BERTDeCS to address the issue of sample scarcity, improving performance, particularly for rare labels with limited positive samples.

To examine the effectiveness, BERTDeCS participated in the MESINESP (Rodriguez-Penagos *et al*. 2020) (Medical Semantic Indexing in Spanish) and MESINESP2 (Gasco *et al*. 2021) competitions and achieved the first place in both. In MESINESP2, BERTDeCS achieved a MiF score of 0.484, representing a 14.5% improvement over the second-best method, PIDNA (0.423). In addition, we conducted extensive experiments and analyses, including grouped analysis according to DeCS frequency and ablation studies. Moreover, experiments on Portuguese biomedical literature suggest that a fully trained BERTDeCS model can transfer to a closely related language without manually indexed Portuguese training data. This result indicates potential applicability beyond Spanish, particularly for related languages that share lexical and biomedical terminology. All these experiments consistently demonstrated the effectiveness and efficiency of BERTDeCS.

## 2 Methods

### 2.1 Overview of BERTDeCS

As shown in Fig. 1, BERTDeCS utilizes M-BERT as its text encoder to process documents in Spanish, English or other languages, and consists of three steps for semantic indexing of biomedical literature: (1) fine-tuning M-BERT using bilingual-based contrastive learning to obtain a domain-adaptive M-BERT tailored for the Spanish literature in VHL, referred to as CL-M-BERT; (2) pre-training the model with CL-M-BERT on millions of English literature from MEDLINE with high-quality manual MeSH semantic indexing to enhance the performance, especially for the rare DeCS codes; and (3) fine-tuning the pre-trained model on Spanish literature from the VHL. The above steps aim to address the challenge of inadequate high-quality indexing in Spanish biomedical literature through contrastive learning and transfer learning.

**Figure 1:**
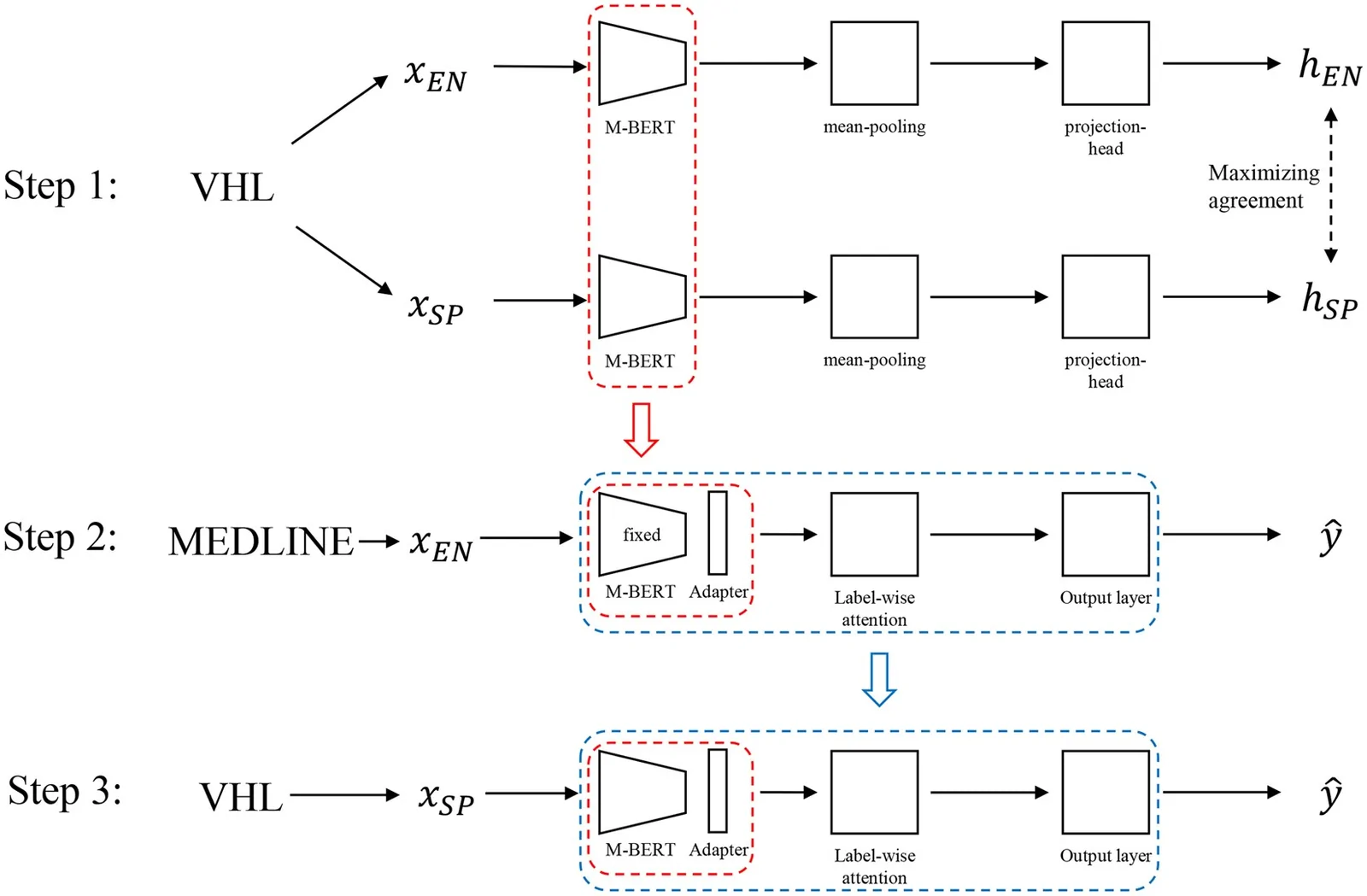
The overall three-step workflow of BERTDeCS. BERTDeCS initially fine-tunes M-BERT using non-English corpora (e.g. VHL for Spanish) via contrastive learning to acquire a domain-adapted encoder. It then pre-trains the model on English corpus with adequate, high-quality semantic indexing. Finally, the pre-trained model is fine-tuned on specific non-English literature. $x_{EN}$ and $x_{SP}$ are input citations in English and Spanish, respectively.

Figure 2 illustrates the detailed network architecture employed in BERTDeCS, drawing inspiration from the effective performance of BERTMeSH (You *et al*. 2021) in MeSH semantic indexing. The architecture comprises three main components: (1) a pre-trained BERT encoder layer; (2) a label-wise attention layer; and (3) fully connected layers and an output layer.

**Figure 2:**
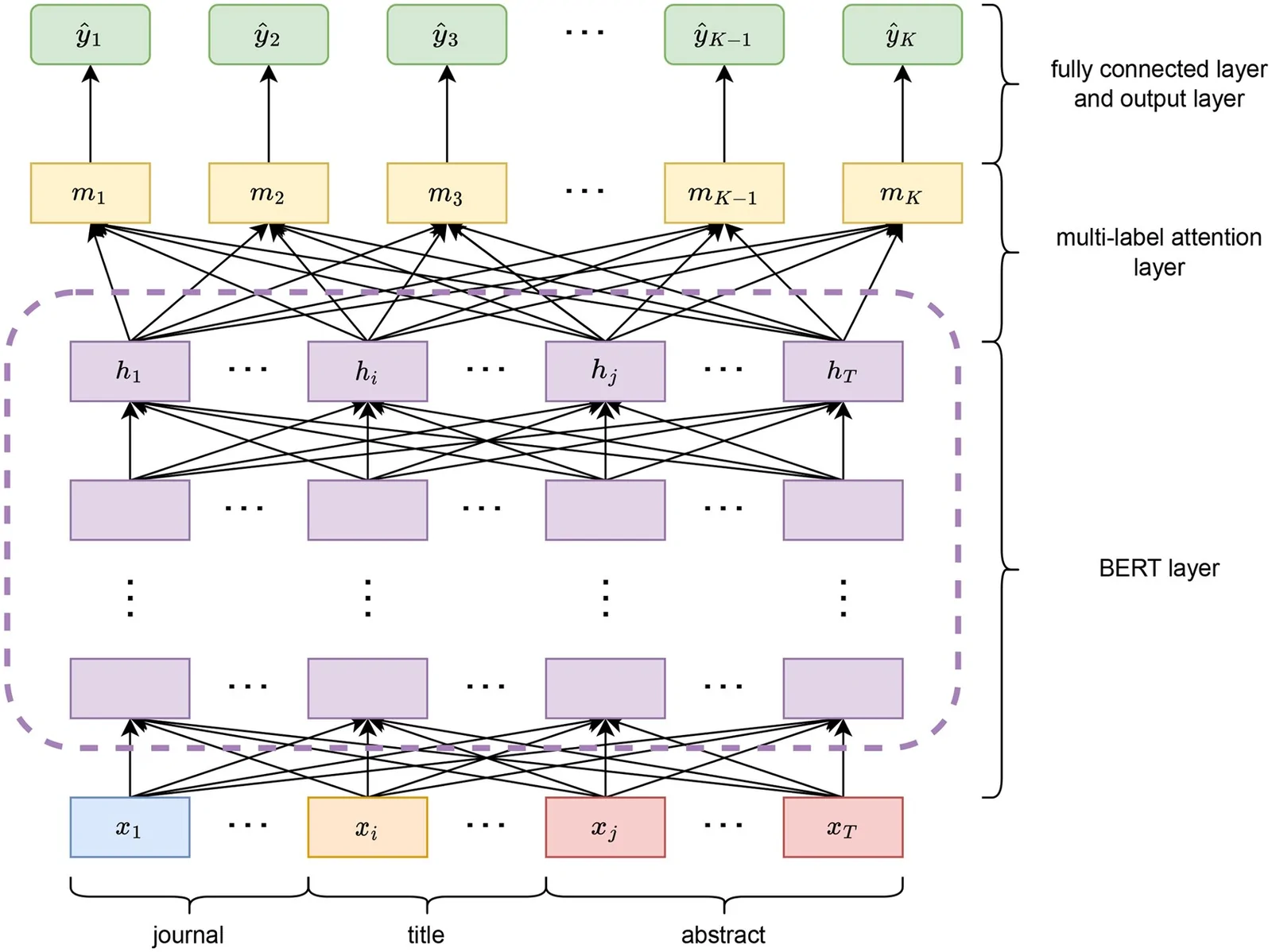
The network architecture of BERTDeCS. The model inputs the concatenation of journal, title, and abstract, and utilizes a BERT layer for encoding. Subsequently, a multi-label attention layer is employed to capture the features of the sentences. Finally, the model utilizes fully connected layers and an output layer to predict confidence scores for each label.

### 2.2 Network architecture of BERTDeCS

For a given citation, we use the name of its journal, title, and abstract as input $X=(x_{1},x_{2},\ldots ,x_{T})$, where $x_{i}$ is the *i*-th token of this given citation. Then we use an M-BERT in the BERT layer as the encoder to obtain its semantic representation as follows:


(1) $$H=\text{BERT}(\theta_{\text{BERT}},X)=(h_{1},h_{2},\ldots ,h_{T})$$

where $H\in R^{T\times n}$, *n* is the hidden size of BERT, $\theta_{\text{BERT}}$ is the weight parameter of BERT Layer, and $h_{i}\in R^{n}$ is the representation of the *i*-th token. After the BERT layer, label-wise attention is used to capture the most relevant parts of each label and obtain different representations of each label as follows:


(2) $$m_{j}=\sum_{i=1}^{T}\alpha_{ij}h_{i},\;\alpha_{ij}=\frac{e^{h_{i}w_{j}}}{\sum_{t=1}^{T}e^{h_{t}w_{j}}},$$

where $w_{j}$ is the attention weight for the *j*-th label and $m_{j}$ is the representation for the *j*-th label. Finally, we use one fully connected layer and one output layer with share weights for all labels, and the predicted probability ${\hat{y}}_{j}$ for the *j*-th label can be computed as follows:


(3) $${\hat{y}}_{j}=\sigma (W_{2}f(W_{1}m_{j}+b_{1})+b_{2}),$$

where $W_{1}\in R^{c\times n}$ and $W_{2}\in R^{1\times c}$ are parameters of the fully connected layer and output layer, respectively, and $b_{1}\in R^{c}$ and $b_{2}\in R$ are bias terms, and *f* is a non-linear (activation) function.

### 2.3 Step 1: bilingual-based contrastive learning

Previous studies on biomedical pre-trained language models, such as BioBERT and MSR BiomedBERT, reported that domain-specific pre-training on biomedical corpora improves performance over general-domain pre-trained models in biomedical text mining tasks (Lee *et al*. 2020, Gu *et al*. 2021). However, these models trained on biomedical texts are limited to supporting English literature. Due to the scarcity of target non-English biomedical literature (e.g. just over 400 000 Spanish literature is available), it is hard to fine-tune the general M-BERT directly using the MLM (masked language model) task, as done for BioBERT, to obtain an ideal model. However, a substantial number of citations in IBECS and LILACS include abstracts in both Spanish and English, offering valuable bilingual resources. An example of a citation of IBECS with both Spanish and English abstracts is shown in Table S2, available as supplementary data at *Bioinformatics* online of the supplement materials. Texts translated into another language can be used as a form of data augmentation for contrastive learning. In addition, the M-BERT we used in BERTDeCS can handle both English and Spanish directly. Therefore, by using unsupervised contrastive learning with this natural and high-quality data augmentation method, we can obtain a more domain-adaptive M-BERT, referred to as CL-M-BERT, tailored for this smaller target domain corpus. Similar to most contrastive learning methods, we use the instance discrimination task (Wu *et al*. 2018), where a positive pair consists of a citation in two languages, and a negative pair consists of any two different citations. The objective function for a positive pair (i,j) we used is NT-Xent (the normalized temperature-scaled cross-entropy loss) (Chen *et al*. 2020), defined as follows:


(4) $$\ell_{i,j}=-\log\frac{\;\text{exp}(\text{cos}(z_{i},z_{j})/\tau )}{{\sum_{k=1}^{2N}1}_{\left[k\neq i\right]}\;\text{exp}(\text{cos}(z_{i},z_{k})/\tau )}$$

where $z_{i}$ is the representation of the *i*-th citation obtained by averaging the output of BERT encoder, $1_{\left[k\neq i\right]}\in \{0,1\}$ is an indicator function evaluating to 1 iff k≠i and τ is a temperature parameter.

### 2.4 Step 2: Pre-training on MEDLINE

In contrast to Spanish, over 10 million English literature in MEDLINE with high-quality manual MeSH indexing is available. Additionally, there is a significant overlap between MeSH and DeCS codes (29 640 out of 34 040). To further enhance performance, particularly for rare DeCS codes with limited positive samples, we employ a transfer learning strategy to leverage the extensive MEDLINE literature with manual MeSH indexing. We first train BERTDeCs with CL-M-BERT on MEDLINE literature and then fine-tune this model on Spanish literature. With M-BERT, we can effortlessly process literature in both Spanish and English. Given the distribution differences between the English literature in MEDLINE and the Spanish literature in VHL, we incorporated adapters (Houlsby *et al*. 2019) in CL-M-BERT, training only the parameters of the adapters to maintain adaptability for the Spanish literature in VHL.

### 2.5 Step 3: training on Spanish literature

We fine-tune the pre-trained model after *Step 2* on Train-ES. As shown in Fig. 1, we initialize the model using weights pre-trained on MEDLINE. During the training process of both *Step 2* and *Step 3*, we use the binary cross-entropy as the objective function as follows:


(5) $$J(\theta )=-\frac{1}{NK}\sum_{i=1}^{N}\sum_{j=1}^{K}\left[y_{ij}\;\text{log}({\hat{y}}_{ij})+(1-y_{ij})\log(1-{\hat{y}}_{ij})\right],$$

where *N* is the number of samples, *K* is the number of labels, ${\hat{y}}_{ij}\in [0,1]$ and $y_{ij}\in \{0,1\}$ are the predicted probability and true value, respectively, for the *i*-th sample and the *j*-th label.

## 3 Results

### 3.1 Data collection

We used MESINESP2-dev (1065 citations) as the validation set to select hyperparameters and MESINESP2-test (491 citations) as the testing set to evaluate the model’s performance. Both MESINESP2-dev and MESINESP2-test are high-quality datasets with manual annotations provided by experts, as invited by the organizers of MESINESP.

For contrastive learning and training, we first collected literature from IBECS (https://ibecs.isciii.es/) and LILACS (http://lilacs.bvsalud.org). We then obtained a total of 3691 citations with DeCS codes and abstracts in Spanish, English, and Portuguese, dated after 2019 and before MESINESP2 (March 2021), as the trilingual testing set (Test-Tri). The training set (Train-ES) comprised 395 245 citations with Spanish abstracts and DeCS codes that were not included in the Test-Tri. For the training set of contrastive learning (train-CL), we used all 302 056 citations having both Spanish and English abstracts. For pre-training on MEDLINE, we downloaded literature in MEDLINE and used the latest 3 million citations with MeSH indexing before 2020.

During the training process, we considered 24 035 DeCS codes appearing in the Train-ES and 21 749 MeSH codes of these DeCS codes when pre-training on MEDLINE. Table 1 shows the detailed statistics of data BERTDeCS used in training and testing. The training and benchmark data is available at https://zenodo.org/records/13824699.

### 3.2 Experimental settings

We implemented BERTDeCS by PyTorch (Paszke *et al*. 2019) and Transformers (Wolf *et al*. 2020). We used M-BERT (Devlin *et al*. 2019) with n=768 and T=512 in the BERT layer, which can handle languages including Spanish, English, Portuguese, and etc. We used the Adam optimizer (Kingma and Ba 2014) with learning rates of 2e−5 for parameters of the BERT layer and 1e−3 for the other parameters, respectively. The batch size we used is 200, and number of epochs is 10. Also, we used a dropout (Srivastava *et al*. 2014) with a drop rate of 0.5 and early stopping to avoid overfitting. For contrastive learning, we used a batch size of 64 and a non-linear projection head. We chose the highest ten labels as our prediction, the average number of labels of the development set(10.59). Table S3 and S5, available as supplementary data at *Bioinformatics* online of the supplement materials shows the ablation test of batch size and fine-tuning the adapter or the entire network, respectively. We ran each model 5 times and chose the best one on development.

### 3.3 Performance evaluation measures

Let *K* be the size of all labels (DeCS), and *N* be the number of instances (literature). Let $y_{i}$ and ${\hat{y}}_{i}\in {\left\{0,1\right\}}^{K}$ be the true and predicted labels for instance *i*, respectively. For performance evaluation, we used the three groups of most common metrics: Micro (precision (MiP), recall (MiR), and F-Measure (MiF)), Macro (precision (MaP), recall (MaP), and F-Measure (MaF)) and Example-Based (on precision (EBP), recall (EBR), and F-Measure (EBF)), which are defined as follows:


(6) $$\text{MiP}=\frac{\sum_{k=1}^{K}\sum_{i=1}^{N}y_{i}^{k}\cdot {\hat{y}}_{i}^{k}}{\sum_{k=1}^{K}\sum_{i=1}^{N}{\hat{y}}_{i}^{k}},$$

(7) $$\text{MiR}=\frac{\sum_{k=1}^{K}\sum_{i=1}^{N}y_{i}^{k}\cdot {\hat{y}}_{i}^{k}}{\sum_{k=1}^{K}\sum_{i=1}^{N}y_{i}^{k}}.$$

(8) $$\text{MiF}=\frac{2\cdot \text{MiP}\cdot \text{MiR}}{\text{MiP}+\text{MiR}}.$$

(9) $${\text{MaP}}_{k}=\frac{\sum_{i=1}^{N}y_{i}^{k}\cdot {\hat{y}}_{i}^{k}}{\sum_{i=1}^{N}{\hat{y}}_{i}^{k}},$$

(10) $${\text{MaR}}_{k}=\frac{\sum_{i=1}^{N}y_{i}^{k}\cdot {\hat{y}}_{i}^{k}}{\sum_{i=1}^{N}y_{i}^{k}}.$$

(11) $$\text{MaF}=\frac{1}{K}\sum_{k=1}^{K}\frac{2\cdot {\text{MaP}}_{k}\cdot {\text{MaR}}_{k}}{{\text{MaP}}_{k}+{\text{MaR}}_{k}}.$$

(12) $${\text{EBP}}_{i}=\frac{\sum_{k=1}^{K}y_{i}^{k}\cdot {\hat{y}}_{i}^{k}}{\sum_{k=1}^{K}{\hat{y}}_{i}^{k}},$$

(13) $${\text{EBR}}_{i}=\frac{\sum_{k=1}^{K}y_{i}^{k}\cdot {\hat{y}}_{i}^{k}}{\sum_{k=1}^{K}y_{i}^{k}}.$$

(14) $$\text{EBF}=\frac{1}{N}\sum_{i=1}^{N}\frac{2\cdot {\text{EBP}}_{i}\cdot {\text{EBR}}_{i}}{{\text{EBP}}_{i}+{\text{EBR}}_{i}}.$$

### 3.4 Experimental results

#### 3.4.1 BERTDeCS accurately predicts DeCS codes for Spanish biomedical literature

We benchmarked BERTDeCS on MESINESP2-test. The competing methods were the other four best methods of MESINESP2: PIDNA, CSSClassifier, Iria-system, and MESINESP_baseline. PIDNA (Huang *et al*. 2021) integrated a transformer-based multi-label classification model along with a trained NER (named entity recognition) system to match the rare DeCS codes. CSSClassifier (García-Pablos *et al*. 2021) encoded the given citation by a pre-trained BERT and built a multi-label classifier following it directly. The Iria-system (Ribadas-Pena *et al*. 2021) utilized a k-NN model based on semantic representations generated by pre-trained BERT, augmented by linguistically motivated index terms such as lemmas, syntactic dependencies, NP chunks, named entities, and keywords. MESINESP2_baseline (Gasco *et al*. 2021), provided by the organizers of MESINESP, consisted of a simple textual lookup system.

As shown in Table 2, BERTDeCS achieved higher scores than all other competing methods in terms of MiF, MaF, and EBF. For example, BERTDeCS achieved the highest MiF score of 0.484, which is 14.5% higher than the second-best method, PIDNA (0.423), and 26.5% higher than the third-best method, CSSClassifier (0.383). A similar trend was observed for EBF, as shown in Table 2. Additionally, BERTDeCS achieved the highest MaF score of 0.397, with MESINESP2_baseline achieving the second-best MaF score of 0.344. BERTDeCS is also the only method whose MaF score surpassed that of the simple lookup system (MESINESP2_baseline), and outperformed PIDNA (0.308), CSSClassifier (0.191), and Iria-system (0.235). These findings indicate that BERTDeCS, leveraging label-wise attention and pre-training on MEDLINE, improves performance for rare DeCS codes (“tail labels”) compared to the other competing methods. Furthermore, as shown in Table S1, available as supplementary data at *Bioinformatics* online of the supplement materials, the results by bootstrap experiment demonstrate that the performance improvement is statistically significant.

**Table 1:** Statistics of data BERTDeCS used.

	Spanish	English	Portuguese	Number
Training Set (Train-ES)	✓	–	–	395 245
Testing Set (Test-Tri)	✓	✓	✓	3691
Contrastive Learning (Train-CL).	✓	✓	×	302 056
MEDLINE	×	✓	×	3 000 000
MESINESP2-dev	✓	–	–	1065
MESINESP2-test	✓	–	–	491
DeCS	–	–	–	34 040
MeSH	–	–	–	29 640

**Table 2:** Performance comparison of BERTDeCS with baselines on MESINESP2-test.

Method	MiF	MiP	MiR	MaF	MaP	MaR	EBF	EBP	EBR
BERTDeCS	**0.484**	**0.508**	**0.462**	**0.397**	0.508	**0.408**	**0.477**	**0.508**	**0.474**
PIDNA	0.423	0.467	0.386	0.308	0.446	0.315	0.412	0.492	0.388
CSSClassifier	0.383	0.462	0.326	0.191	0.443	0.192	0.373	0.500	0.329
Iria-system	0.373	0.419	0.335	0.235	**0.535**	0.233	0.366	0.425	0.340
MESINESP_baseline	0.288	0.234	0.375	0.344	0.372	0.379	0.284	0.245	0.384

The highest values are bolded and the second highest values are underlined.

#### 3.4.2 BERTDeCS outperforms other competing methods across different DeCS groups

Based on the number of occurrences of DeCS codes in the training set (Train-ES), we divide the DeCS codes into five groups: [1, 100), [100, 500), [500, 1000), [1000, 5000), and [5000, +∞). The group [1, 100) means that each DeCS code in this group has a number of occurrences between 1 (including) and 100 (excluding) in Train-ES. Figure 3a shows the distributions of DeCS codes and DeCS indexing of these five groups. Although the group [1, 100) accounts for over 87% of all distinct DeCS codes, it accounts for around 15% of the total DeCS indexing. It indicates that the handling approach of these “long-tailed” labels directly impacts the performance of models, especially MaF.

**Figure 3:**
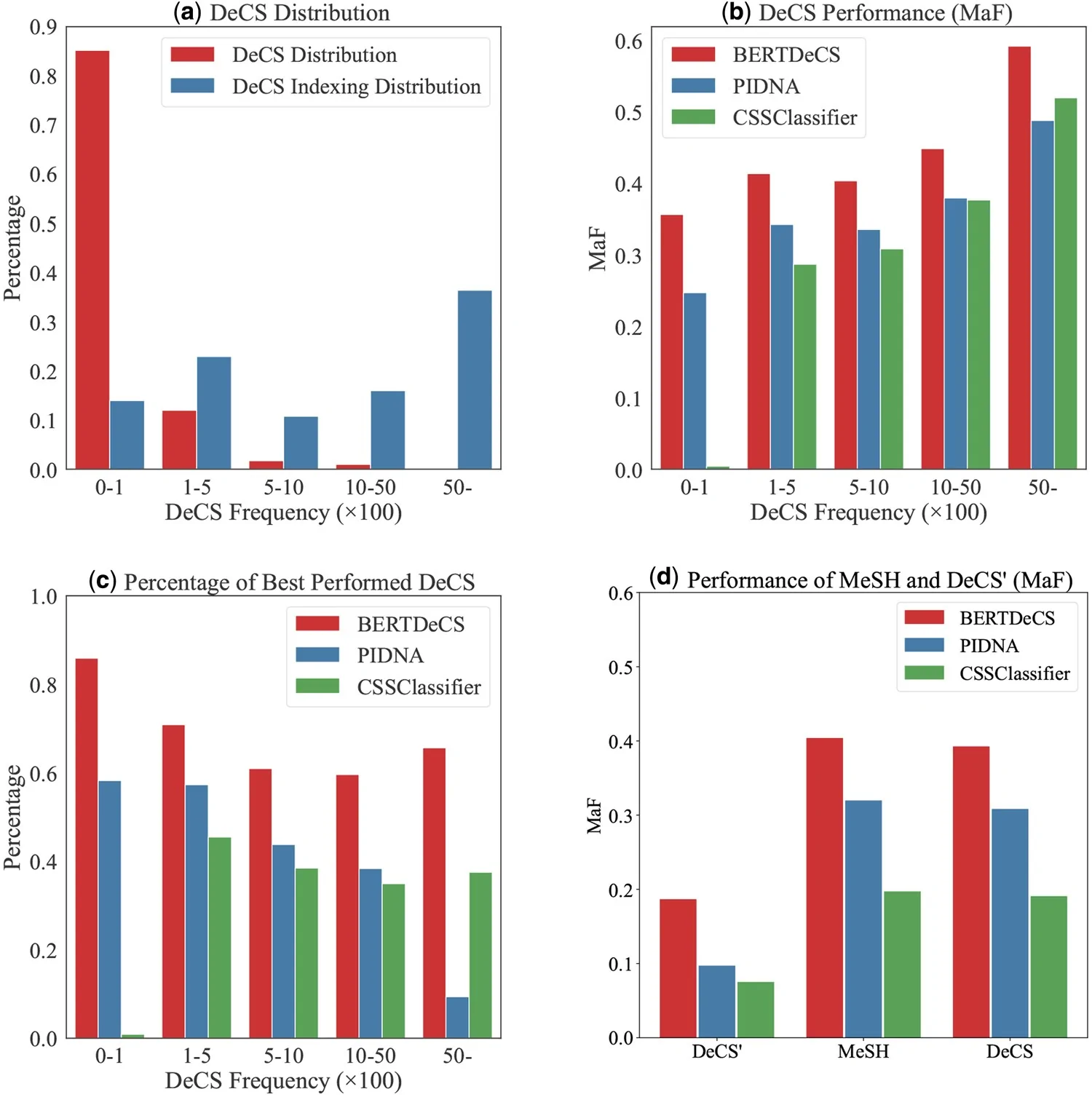
(a) DeCS distribution and DeCS indexing distribution in the train-ES. (b) MaF performance of BERTDeCS, PIDNA and CSSClassifier among different DeCS groups. (c) Percentage of best performed DeCS of BERTDeCS, PIDNA, and CSSClassifier among different DeCS groups. (d) MaF performance of BERTDeCS, PIDNA and CSSClassifier on DeCS’, MeSH and DECS terms.

We benchmarked BERTDeCS against PIDNA and CSSClassifier on MESINESP2-test using MaF for each group in Fig. 3b. Figure 3c shows the distributions of these three methods regarding the best predictive methods (allowing ties) on each DeCS group. BERTDeCS consistently outperformed both PIDNA and CSSClassifier, particularly in the less frequent groups. This highlights the advantage of BERTDeCS over PIDNA and CSSClassifier regardless of the frequency of DeCS. In this comparison, the CSSClassifier performs significantly worse than PIDNA for the least frequent group but outperforms PIDNA for the most frequent group in terms of MaF. The explanation could be that PIDNA integrates a trained NER system to match rare DeCS codes, providing a valuable advantage for handling infrequently occurring DeCS codes.

On the other hand, DeCS codes can also be divided into two types: those originated from MeSH terms (MeSH) and newly added area-related specific DeCS codes (DeCS’). Figure 3d provides the performance (MaF) of BERTDeCS, PIDNA and CSSClassifier, respectively, when considering DeCS’, MeSH and DeCS terms. BERTDeCS achieved higher performance than the other methods in both types and showed less degradation for specific DeCS codes. Due to the pre-training on MEDLINE, BERTDeCS inherited the powerful indexing ability considering those DeCS codes originated from MeSH terms. Moreover, leveraging the M-BERT encoder and the contrastive learning step allows BERTDeCS to effectively transfer comprehension from MeSH terms to new DeCS codes, showcasing commendable indexing performance.

#### 3.4.3 BERTDeCS transfers to Portuguese biomedical documents under zero-shot setting

To evaluate the performance of BERTDeCS for biomedical documents in Portuguese, we used a test set consisting of biomedical literature available in English, Spanish, and Portuguese (Test-Tri). As shown in Table 3, even though Portuguese was not present in the training and pre-training datasets of BERTDeCS, using Portuguese as input still resulted in a MiF of 0.501, which is only about 5% lower than the MiF of 0.531 achieved with Spanish, and around 4% lower than the MiF of 0.523 achieved with English. These results suggest that BERTDeCS has some ability to transfer to Portuguese, a language closely related to Spanish and sharing substantial biomedical terminology.

**Table 3:** Performance comparison of BERTDeCS on test-ES, test-EN, and test-PT.

Language	MiF	MiP	MiR	MaF	MaP	MaR	EBF	EBP	EBR
ES	**0.531**	0.618	**0.465**	0.292	0.542	0.291	**0.504**	0.624	**0.471**
EN	0.523	0.614	0.456	**0.298**	0.528	**0.297**	0.496	0.624	0.461
PT	0.501	**0.636**	0.413	0.244	**0.568**	0.237	0.472	**0.648**	0.417

The highest values are bolded.

#### 3.4.4 The number of recommended labels affects the performance

To assess the impact of the number of recommended labels on prediction performance, we compared BERTDeCS when recommending 10 labels against recommending the exact number of correct labels. The number 10 represents the average number of labels in the MESINESP2-dev and is also the default setting for BERTDeCS. As shown in Table 4, recommending the correct number of labels improved MiF and MaF by 3.9% and 6.8%, respectively, compared to recommending a fixed number of labels. This gap is primarily due to differences in recall. This indicates that predicting the correct number of labels or adjusting the score threshold can further enhance the model’s predictive accuracy.

**Table 4:** Comparison of BERTDeCS with a fixed number (10) and a true number of recommended labels.

Method	MiF	MiP	MiR	MaF	MaP	MaR	EBF	EBP	EBR
BERTDeCS_10_	0.484	**0.508**	0.462	0.397	**0.508**	0.408	0.477	**0.508**	0.474
BERTDeCS_True_	**0.502**	0.502	**0.502**	**0.424**	0.494	**0.441**	**0.485**	0.485	**0.485**

The highest values are bolded.

#### 3.4.5 Ablation analysis of contrastive learning and MEDLINE pre-training

As shown in Fig. 1, BERTDeCS involves three steps: (1) pre-training M-BERT with bilingual-based contrastive learning (CL) to get a domain-adapted encoder (referred to as CL-M-BERT); (2) pre-training (PT) on MEDLINE; and (3) fine-tuning (FT) on Spanish literature with DeCS codes indexing. To examine the effectiveness of CL and PT in BERTDeCS, we compared BERTDeCS with CSSClassifier (which can be considered as a variant of BERTDeCS without using pre-training and label-wise attention), EriBERTa as a domain-specific Spanish–English biomedical/clinical encoder, and three variants of BERTDeCS: BERTDeCS${\;}_{\text{FT}}$ (BERTDeCS without CL and PT), BERTDeCS${\;}_{\text{CL}+\text{FT}}$ (BERTDeCS without PT), and BERTDeCS${\;}_{\text{PT}+\text{FT}}$ (BERTDeCS without CL). For convenience, we refer to BERTDeCS with BERTDeCS${\;}_{\text{CL}+\text{PT}+\text{FT}}$ in this section.

Table 5 presents the results of the ablation study, highlighting the performance of various configurations of BERTDeCS. BERTDeCS${\;}_{\text{FT}}$ achieved a MiF of 0.468, MaF of 0.360, and EBF of 0.459, outperforming CSSClassifier by 22.2%, 88.5%, and 23.1%, respectively (CSSClassifier scores: 0.383, 0.191, and 0.373). This demonstrates the efficacy of label-wise attention in capturing the most pertinent text segments for each label, particularly for rare DeCS codes. The EriBERTa-based classifier achieved a MiF of 0.473, MaF of 0.375, and EBF of 0.467, slightly outperforming BERTDeCS${\;}_{\text{FT}}$ and confirming that EriBERTa is a strong domain-specific encoder for Spanish biomedical semantic indexing. However, BERTDeCS${\;}_{\text{CL}+\text{PT}+\text{FT}}$ achieved higher overall performance, suggesting that adapting M-BERT and transferring large-scale MEDLINE indexing knowledge provide complementary benefits beyond directly using a domain-specific bilingual encoder. BERTDeCS${\;}_{\text{PT}+\text{FT}}$ recorded a MiF of 0.477, MaF of 0.393, and EBF of 0.470, representing improvements of 1.9%, 9.2%, and 2.4% over BERTDeCS${\;}_{\text{FT}}$ (0.468, 0.360, and 0.459). These findings highlight the advantages of pre-training on MEDLINE (*Step 2*), which benefits from the millions of English literature with high-quality semantic indexing in MEDLINE. Moreover, BERTDeCS${\;}_{\text{CL}+\text{PT}+\text{FT}}$ achieved the highest performance across all metrics, with a MiF of 0.484, compared with 0.477 for BERTDeCS${\;}_{\text{PT}+\text{FT}}$ without contrastive learning. This corresponds to a modest improvement of 1.5% in MiF. These results indicate that contrastive learning provides an additional but limited gain when combined with MEDLINE pre-training, and should be viewed as an auxiliary domain-adaptation step rather than the sole driver of BERTDeCS performance. In addition, BERTDeCS${\;}_{\text{CL}+\text{FT}}$ achieved nearly identical performance to BERTDeCS${\;}_{\text{FT}}$. This can be attributed to the fact that the contrastive learning pre-training model, CL-M-BERT, was trained on Train-CL and then fine-tuned on Train-ES, with approximately 75% of the Train-CL dataset overlapping with Train-ES. In such cases, where there is substantial overlap between the pre-training and fine-tuning datasets, pre-training does not contribute to performance improvement. In contrast, in BERTDeCS${\;}_{\text{CL}+\text{PT}+\text{FT}}$, the CL-M-BERT was fine-tuned on MEDLINE, which has no overlap with Train-CL. As a result, contrastive learning provides its main benefit when combined with transfer pre-training on MEDLINE, where it serves as an encoder adaptation step before learning from large-scale English annotations.

**Table 5:** Ablation study and encoder comparison of BERTDeCS.

Method	MiF	MiP	MiR	MaF	MaP	MaR	EBF	EBP	EBR
CSSClassifier	0.383	0.462	0.326	0.191	0.443	0.192	0.373	0.500	0.329
EriBERTa	0.473	0.497	0.452	0.375	0.506	0.384	0.467	0.497	0.465
BERTDeCS_FT_	0.468	0.491	0.446	0.360	0.501	0.370	0.459	0.491	0.455
BERTDeCS_CL+FT_	0.467	0.490	0.446	0.359	0.485	0.370	0.460	0.490	0.457
BERTDeCS_PT+FT_	0.477	0.501	0.456	0.393	0.505	0.400	0.470	0.501	0.467
BERTDeCS_CL+PT+FT_	**0.484**	**0.508**	**0.462**	**0.397**	**0.508**	**0.408**	**0.477**	**0.508**	**0.474**

*Abbreviations:* CL, contrastive learning; PT, MEDLINE pre-training; FT, fine-tuning on Spanish DeCS annotations.

The highest values are bolded.

To visually demonstrate the improvement in literature representation brought by contrastive learning, we selected two journals with the largest number of document samples in the training set (*Nutrición Hospitalaria* and *Revue Neurologique* [*Hospital Nutrition* and *Neurological Review* in English]) and used T-SNE to visualize all documents from these journals for both M-BERT and CL-M-BERT, as shown in Fig. 4a and b. Note that documents from the same journal tend to cover similar topics, whereas those from different journals are expected to have distinct topics. Figure 4a and b indicate that, compared with M-BERT, CL-M-BERT achieves better document representation capabilities through contrastive learning, which can distinguish the documents from different journals more clearly.

**Figure 4:**
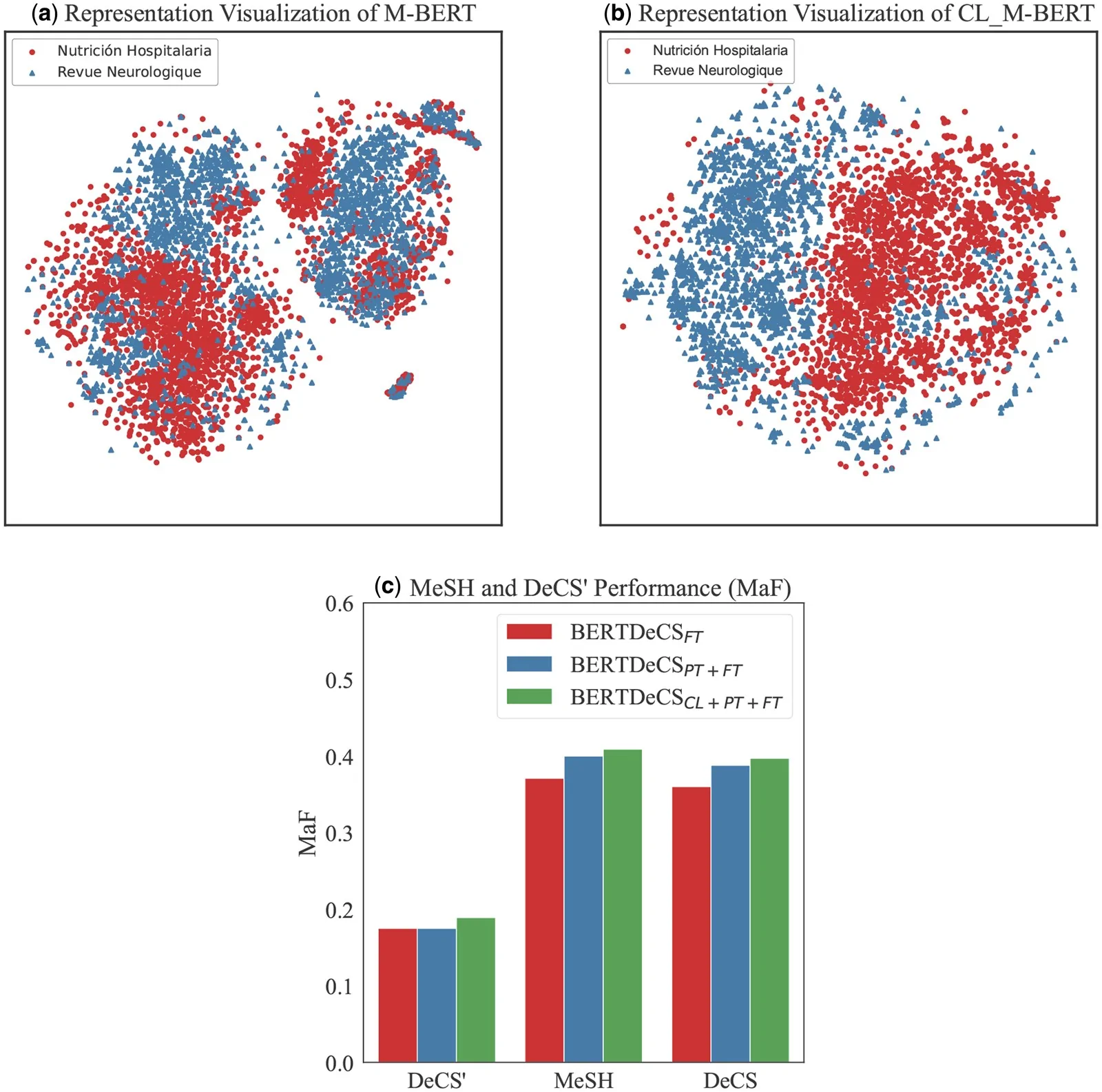
(a, b) Representation visualization of articles in *Nutrición Hospitalaria* (*Hospital Nutrition*, including 3209 articles) and *Revue Neurologique* (*Neurological Review*, including 2517 articles). (c) MaF performance of BERTDeCS${\;}_{\text{FT}}$, BERTDeCS${\;}_{\text{PT}+\text{FT}}$ and BERTDeCS${\;}_{\text{CL}+\text{PT}+\text{FT}}$ on DeCS’, MeSH and DeCS terms.

To further validate the impact of each step in BERTDeCS, we compared BERTDeCS${\;}_{\text{FT}}$, BERTDeCS${\;}_{\text{PT}+\text{FT}}$, and BERTDeCS${\;}_{\text{CL}+\text{PT}+\text{FT}}$ in terms of their performance (MaF) on DeCS’ and MeSH terms, respectively. As shown in Fig. 4c, BERTDeCS${\;}_{\text{CL}+\text{PT}+\text{FT}}$ achieved the best performance on both MeSH and DeCS’ terms. In contrast, BERTDeCS${\;}_{\text{PT}+\text{FT}}$ improved over BERTDeCS${\;}_{\text{FT}}$ on MeSH terms, with only slight improvement on DeCS’ terms. This indicates that the pre-training using MEDLINE in Step 2 mainly enhances the prediction performance on MeSH labels. Furthermore, compared to BERTDeCS${\;}_{\text{PT}+\text{FT}}$, BERTDeCS${\;}_{\text{CL}+\text{PT}+\text{FT}}$ further improved MaF on both DeCS’ and MeSH terms. This suggests that contrastive learning helps adapt the encoder representation, although its contribution is smaller than that of MEDLINE pre-training. Table S4, available as supplementary data at *Bioinformatics* online of the supplement materials further shows the ablation study of BERTDeCS in Portuguese under zero-shot settings.

Overall, these results suggest that the final performance of BERTDeCS comes from the integration of label-wise attention, MEDLINE pre-training, supervised fine-tuning on Spanish DeCS annotations, and contrastive learning, with contrastive learning providing an additional but modest contribution. Supplement materials show a detailed case study of different steps in BERTDeCS.

#### 3.4.6 Comparison with DeCS Finder AI and LLM prompting baselines

To provide an initial assessment of the current relevance of BERTDeCS, we further compared it with DeCS Finder AI and LLM prompting baselines. DeCS Finder AI is an official automatic DeCS annotation system developed by BIREME (https://decsfinder.bvsalud.org). For the LLM baseline, we used Qwen2.5-7B (*Yang et al.* 2024) to predict the DeCS descriptors from the Spanish title and abstract of each article. We evaluated 0-shot, 1-shot, 2-shot, and 3-shot prompting settings. To reduce the influence of demonstration selection, each few-shot setting was repeated five times using different examples, and the final results were reported as the average across runs. More details are provided in the supplementary materials.

As shown in Table 6, BERTDeCS achieved the best overall performance in MiF, MaF, and EBF, obtaining 0.484, 0.397, and 0.477, respectively. DeCS Finder AI achieved a MiF of 0.361 and a MaF of 0.389. Qwen2.5-7B achieved MiF scores from 0.319 to 0.351 under 0-shot to 3-shot prompting settings. These results show that BERTDeCS remains effective for controlled DeCS descriptor assignment when compared with DeCS Finder AI and LLM prompting baselines. Nevertheless, this comparison is an initial assessment rather than an exhaustive evaluation of all prompting strategies, larger open-source models, or proprietary generative models.

**Table 6:** Comparison of BERTDeCS with DeCS Finder AI and Qwen2.5-7B prompting baselines.

Method	MiF	MiP	MiR	MaF	MaP	MaR	EBF	EBP	EBR
DeCS Finder	0.361	0.318	0.417	0.389	0.330	0.444	0.355	0.318	0.431
Qwen-0shot	0.319	0.428	0.255	0.265	0.478	0.270	0.317	0.446	0.268
Qwen-1shot	0.327	0.443	0.260	0.242	0.479	0.246	0.323	0.472	0.270
Qwen-2shot	0.347	0.467	0.277	0.239	0.487	0.242	0.341	0.495	0.284
Qwen-3shot	0.351	0.479	0.278	0.233	0.488	0.235	0.345	**0.509**	0.284
BERTDeCS	**0.484**	**0.508**	**0.462**	**0.397**	**0.508**	**0.408**	**0.477**	0.508	**0.474**

The highest values are bolded.

## 4 Discussion and conclusion

Spanish is widely used worldwide, and a large amount of biomedical literature is written in Spanish. Therefore, automatic semantic indexing of Spanish biomedical literature is of great significance. However, Spanish biomedical literature lacks sufficient high-quality manually annotated training data and a domain-adaptive large-scale pre-trained language model. To overcome the challenges, we have developed a novel deep learning-based model, BERTDeCS, with bilingual-based contrastive learning and pre-training. BERTDeCS employs a multi-label attention mechanism to capture the most relevant parts of each label and obtain their respective representations. By using M-BERT as its text encoder rather than BERT, BERTDeCS can process English and Spanish and may be applicable to other non-English languages supported by M-BERT. Besides, BERTDeCS fine-tunes M-BERT by bilingual-based contrastive learning to obtain a more domain-adaptive text encoder for non-English biomedical literature. Furthermore, BERTDeCS can leverage substantial quantities of high-quality manually annotated English biomedical literature from MEDLINE through transfer learning, thereby alleviating the data quality limitations associated with non-English biomedical literature.

BERTDeCS and its earlier version participated and achieved the best performance in both MESINESP and MESINESP2 competitions, which are semantic indexing of Spanish biomedical literature tasks. Additionally, we conducted extensive experiments to validate the effectiveness of Step 1 (bilingual contrastive learning) and Step 2 (pre-training on MEDLINE), showing that MEDLINE pre-training provides a substantial improvement, while contrastive learning provides an additional but modest contribution as an encoder adaptation step. Despite BERTDeCS achieving the best results among competing methods, the performance of semantic indexing for Spanish biomedical literature still lags significantly behind that of English due to the limitations in the scale and quality of annotated data.

While BERTDeCS is trained and mainly tested on Spanish biomedical literature in this study, its framework can be extended to biomedical indexing in other non-English languages. The Portuguese experiments suggest that BERTDeCS can transfer to a closely related language when manually indexed target-language data are unavailable. An interesting extension would be to evaluate BERTDeCS on a more diverse set of typologically distant languages. In addition, since BERTDeCS does not leverage descriptive label information, it cannot perform predictions for labels that are absent from the training set, which presents a potential area for improvement.

## Supplementary Material

btag794_Supplementary_Data
