# Supplementary material for: Large-scale semantic indexing of Spanish biomedical literature using contrastive transfer learning
Source: Bioinformatics. 2026 Oct 24;42(11):btag794. doi: 10.1093/bioinformatics/btag794 (PMC13750898; doi:10.1093/bioinformatics/btag794)
Supplement: Supplementary file 1 [file btag794_supplementary_data.pdf]

Supplementary material of Large-scale semantic indexing of Spanish biomedical literature using contrastive transfer learning

Ronghui You[1], Tianyang Huang[2], Ziye Wang[3], Yuxuan Liu[4], Hong Zhou[5], Shanfeng Zhu[2,4,6,7*]

[1]School of Statistics and Data Science, LPMC, Nankai University.
[2]Institute of Science and Technology for Brain-Inspired Intelligence and MOE Frontiers Center for Brain Science, Fudan University.
[3]School of Mathematical Sciences and LPMC, Nankai University.
[4]Shanghai Key Lab of Intelligent Information Processing and Shanghai Institute of Artificial Intelligence Algorithm, Fudan University.
[5]Head Office, KnowledgeWorks Global Ltd., Glen Allen, VA 23059, USA.
[6]Key Laboratory of Computational Neuroscience and Brain-Inspired Intelligence (Fudan University), Ministry of Education.
[7]Zhangjiang Fudan International Innovation Center.

*Corresponding author(s). E-mail(s): zhusf@fudan.edu.cn;

1 Bootstrap experiment

We repeated bootstrap with replacement 100 times on MESINESP2-test to generate 100 data sets and then conducted paired *t*-test over 100 trials to examine the statistical significance of performance improvement between BERTDeCS and three competing methods: PIDNA, CSSClassifier, and Iria-system. Table S1 reports the statistical results of the performance comparison of BERTDeCS and three competing methods. BERTDeCS achieved the best MiF of 0.483, MaF of 0.395, and EBF of 0.476.

Table S1 Statistical significance test by bootstrapping on MESINESP2-test.

Method	MiF	MiP	MiR	MaF	MaP	MaR	EBF	EBP	EBR
BERTDeCS	**0.483**	**0.507**	**0.462**	**0.395**	0.508	**0.407**	**0.476**	**0.507**	**0.473**
	-	-	-	-	-	-	-	-	-
PIDNA	0.422	0.466	0.385	0.312	0.451	0.319	0.411	0.490	0.387
	5.9e-106	6.9e-78	4.7e-110	1.2e-113	-	4.2e-110	8.9e-107	1.9e-40	2.3e-112
CSSClassifier	0.382	0.461	0.327	0.199	0.444	0.202	0.373	0.498	0.329
	1.1e-124	1.4e-83	3e-134	1.2e-141	-	8.2e-143	5.5e-125	1.3e-20	1.7e-132
Iria-system	0.373	0.419	0.335	0.236	**0.529**	0.237	0.366	0.424	0.341
	2.6e-125	2e-110	1.3e-132	3.3e-139	-	6e-142	3e-124	4.6e-107	2.6e-129

2 Example of IBECS citation

Table S2 shows an example of a citation of IBCES (ibc-14982) which has Spanish and English abstracts. We can see that the translation by manual is highly precise.

3 Impact of batch size of contrastive learning

For examining the impact of a batch size of pre-training by contrastive learning in BERTDeCS, we tried three different batch sizes of contrastive learning: 16, 32, and 64. Table S3 reports performance results of different batch sizes. BERTDeCS, with a batch size of 64 of contrastive learning, achieved the highest MiF of

Table S2 An example of citation ibc-14982 of IBECS.

ID	ibc-14982
Title	Pharmacy services in psychiatry outpatients. Interaction physician-pharmacist
Abstract (Spanish)	El objetivo del trabajo es demostrar las ventajas de la interrelación profesional entre el médico y el farmacéutico en un programa de Atención Farmacéutica en pacientes ambulatorios con Trastornos Psiquiátricos. Ademas de ser una modalidad de trabajo necesaria en el abordaje de cualquier patología, es critica en estos pacientes, existiendo varios puntos en los cuales una falta de coordinación puede hacer fracasar el tratamiento (AU)
Abstract (English)	The objective of this work is to show the advantage of the interaction between physicians and pharmacists in a psychiatric outpatients Pharmaceutical Care program. Besides it should be a normal practice in every pathology, is critical in these patients because there are several subjects that if are not coordinate will lead to a treatment failure (AU)

0.484, MaF of 0.397, and EBF of 0.477, following batch sizes of 32 and 16. These results suggest that using a larger batch size with more negative samples in contrastive learning obtains a higher quality encoder.

Table S3 Performance comparison on test set of MESINESP2.

Batch size of CL	MiF	MiP	MiR	MaF	MaP	MaR	EBF	EBP	EBR
16	0.475	0.499	0.454	0.379	0.506	0.388	0.466	0.499	0.462
32	0.479	0.503	0.458	0.388	**0.523**	0.398	0.471	0.503	0.468
64	**0.484**	**0.508**	**0.462**	**0.397**	0.508	**0.408**	**0.477**	**0.508**	**0.474**

4 Ablation study under zero-shot settings

As shown in the Table S4, we have reported the zero-shot performance for Portuguese of $\text{BERTDeCS}_{\text{FT}}$, $\text{BERTDeCS}_{\text{PM+FT}}$, $\text{BERTDeCS}_{\text{CL+PM+FT}}$. We can see a similar trend to MESINESP2-test.

Table S4 Ablation study of BERTDeCS in Portuguese under zero-shot settings.

Method	MiF	MiP	MiR	MaF	MaP	MaR	EBF	EBP	EBR
$\text{BERTDeCS}_{\text{FT}}$	0.486	0.648	0.389	0.206	0.565	0.198	0.455	0.650	0.388
$\text{BERTDeCS}_{\text{PT+FT}}$	0.498	**0.658**	0.400	0.235	**0.588**	0.225	0.468	**0.663**	0.400
$\text{BERTDeCS}_{\text{CL+PT+FT}}$	**0.501**	0.636	**0.413**	**0.244**	0.568	**0.237**	**0.472**	0.648	**0.471**

5 Comparison between fine-tuning the adapter and the entire newtork

In step 2, we only fine-tuned the adapter. We also attempted fine-tuning the adapter only in step 3. As shown in Table S5, the performance of $\text{BERTDeCS}_{\text{adapter}}$ remarkably decreases when only the adapter is fine-tuned.

Table S5 Comparison between BERTDeCS fine-tuning only the adapter and fine-tuning the entire network.

Method	MiF	MiP	MiR	MaF	MaP	MaR	EBF	EBP	EBR
$\text{BERTDeCS}_{\text{adapter}}$	0.471	0.495	0.450	0.380	0.509	0.387	0.463	0.495	0.460
BERTDeCS	**0.484**	**0.508**	**0.462**	**0.397**	**0.508**	**0.408**	**0.477**	**0.508**	**0.474**

6 Case Study

Here we present predictions of BERTDeCS on a sample Spanish article with ID=ibc-ET5-1543 in Table S6. The first three rows of the table include the title in Spanish/English, the abstract in Spanish/English, and the manual annotated DeCS codes. The last six rows display the predicted DeCS codes from CSSClassifier,

IRIA, PIDNA, BERTDeCS$_{\mathrm{FT}}$, BERTDeCS$_{\mathrm{PT+FT}}$, and BERTDeCS$_{\mathrm{CL+PT+FT}}$. Correctly predicted DeCS terms are highlighted in bold.

As shown in Table S6, BERTDeCS$_{\mathrm{CL+PT+FT}}$ achieves the highest F1 score of 0.667, followed by BERTDeCS$_{\mathrm{PT+FT}}$ of 0.571 and BERTDeCS$_{\mathrm{FT}}$ of 0.476. Notably, both BERTDeCS$_{\mathrm{CL+PT+FT}}$ and BERTDeCS$_{\mathrm{PT+FT}}$ correctly predict a rare MeSH term, **'Infecciones Relacionadas con Pr ´otesis'** (highlighted in blue), which means prosthesis related infections, and is not predicted by BERTDeCS$_{\mathrm{FT}}$. This demonstrates that after transfer learning on MEDLINE, BERTDeCS enhances the semantic representation of literature, resulting in a stronger representational capability for rare terms with low frequency. Besides, BERTDeCS$_{\mathrm{CL+PT+FT}}$ correctly predicts the code **'Farmacocinética'** (highlighted in red), which is not predicted by both BERTDeCS$_{\mathrm{PT+FT}}$ and BERTDeCS$_{\mathrm{FT}}$. The term **'Farmacocinética'** refers to literature that reports on pharmacokinetics, which requires the classifier to comprehend the core semantics of the article to predict the correct code. This indicates that BERTDeCS, after undergoing contrastive learning, has acquired a stronger and more adaptive encoder, enabling the model to better understand the overall gist of the literature. In addition, we have identified descriptions in the literature that are closely related to these terms (highlighted in light blue and light red respectively). We found that when these descriptions are removed from the input text, BERTDeCS is correspondingly unable to predict the associated terms. This process resembles the way experts manually annotate literature, indicating that the model has acquired related knowledge during training.

7 AI Approaches Settings

To further investigate recent AI-assisted and generative approaches for multilingual biomedical semantic indexing, we additionally evaluated both the official DeCS Finder AI system developed by BIREME and a prompt-based large language model baseline.

For the DeCS Finder AI experiments, since no official batch-processing API is currently publicly available, we implemented a browser-based automation pipeline to process the MESINESP test set. Specifically, the Spanish title and abstract of each article were automatically submitted through the web interface, and the exported indexing results were collected. Among the exported outputs, we used the "Suggested by AI" descriptors as the final predictions for evaluation.

For the generative AI baseline, we used Qwen2.5-7B-Instruct to directly generate DeCS-related descriptors from the Spanish title and abstract using prompt-based instruction following. The detailed prompt template is provided below. Since the model outputs free-text biomedical terms rather than standardized DeCS identifiers, we further applied a normalization and mapping pipeline. First, the generated terms were normalized through basic text preprocessing. Subsequently, synonym matching was performed against the official DeCS terminology dictionary to obtain the corresponding DeCS identifiers. Terms that could not be successfully matched to any valid DeCS entry were discarded. For few-shot settings, different demonstration examples were sampled across repeated runs, and the final reported performance was averaged over five independent experiments.

Table S6 An example for indexing results through BERTDeCS.

Title (Spanish/English)	Monitorización farmacocinética ambulatoria de teicoplanina por infección crónica de prótesis de rodilla / Outpatient pharmacokinetic monitoring of teicoplanin for chronic knee prosthesis infection
Abstract (Spanish/English)	Presentamos el caso de un paciente con infección crónica de prótesis de rodilla, sometido a múltiples intervenciones quirúrgicas y tratamientos antibióticos previos. La indicación de Traumatología es tratamiento antibiótico de supresión crónico, contraindicando nueva intervención quirúrgica. En esta situación, se constata que la infección está causada por una mezcla de cepas de Staphylococus epidermidis con heterorresistencia con sensibilidad únicamente a linezolid y a glupopéptidos. Se descartó el uso crónico de linezolid por razones de toxicidad hematológica y neuropatía óptica y periférica descritos en tratamientos superiores a 28 días. Se propuso teicoplanina por su buen perfil farmacocinético, que la hace óptima para regímenes de administración ambulatoria. El seguimiento del tratamiento se basó en la monitorización farmacocinética de los niveles valle de teicoplanina, y ajuste de dosis en función de los mismos. Se realizó seguimiento de parámetros de inflamación, hematológicos y de función renal para monitorizar la efectividad y toxicidad del tratamiento. El paciente presentó durante el periodo de seguimiento (18 meses) una evolución clínica satisfactoria sin toxicidad destacable. En este sentido, en pacientes seleccionados, teicoplanina administrada por unidades ambulatorias de administración de antibióticos puede resultar útil en el tratamiento de infecciones crónicas de prótesis articulares. / We present the case of a patient with a chronic knee prosthesis infection, who underwent multiple surgical interventions and prior antibiotic treatments. The indication from Traumatology was chronic antibiotic suppression therapy, with surgical re-intervention contraindicated. In this situation, it was found that the infection was caused by a mixture of Staphylococcus epidermidis strains with heteroresistance, sensitive only to linezolid and glycopeptides. The chronic use of linezolid was ruled out due to hematologic toxicity and optical and peripheral neuropathy described in treatments longer than 28 days. Teicoplanin was proposed due to its good pharmacokinetic profile, which makes it optimal for outpatient administration regimens. The treatment follow-up was based on pharmacokinetic monitoring of teicoplanin trough levels, with dosage adjustments accordingly. Monitoring of inflammation, hematologic, and renal function parameters was performed to track the effectiveness and toxicity of the treatment. During the 18-month follow-up period, the patient showed satisfactory clinical progress without notable toxicity. In this context, teicoplanin, administered by outpatient antibiotic administration units, may be useful in treating chronic prosthetic joint infections in selected patients.
DeCS	Farmacocinética, Infecciones, Humanos, Pruebas de Función Renal, Teicoplanina, Infecciones Relacionadas con Prótesis, Prótesis de la Rodilla, Masculino, Staphylococcus epidermidis, Prótesis Articulares, Anciano de 80 o más Años
CSSClassifier (F1: 0.471)	**Humanos**, **Prótesis de la Rodilla**, **Masculino**, Artroplastia de Reemplazo de Rodilla, **Infecciones Relacionadas con Prótesis**, Anciano
IRIA (F1: 0.286)	Antibacterianos, **Teicoplanina**, **Humanos**, **Masculino**, Femenino, Resultado del Tratamiento, Cuidados Posteriores, Efectividad, Rodilla, Naciones Unidas
PIDNA (F1: 0.476)	**Humanos**, **Masculino**, **Prótesis de la Rodilla**, Anciano, Persona de Mediana Edad, Artroplastia de Reemplazo de Rodilla, **Anciano de 80 o más Años**, Linezolid, Enfermedades del Nervio Óptico, **Teicoplanina**
$\text{BERTDeCS}_{\text{FT}}$ (F1: 0.476)	**Teicoplanina**, **Humanos**, **Masculino**, **Prótesis de la Rodilla**, Artroplastia de Reemplazo de Rodilla, Antibacterianos, **Staphylococcus epidermidis**, Adulto, Monitoreo de Drogas, Atención Ambulatoria
$\text{BERTDeCS}_{\text{PT+FT}}$ (F1: 0.571)	**Humanos**, **Teicoplanina**, **Masculino**, **Prótesis de la Rodilla**, Antibacterianos, **Infecciones Relacionadas con Prótesis**, Artroplastia de Reemplazo de Rodilla, Enfermedad Crónica, **Staphylococcus epidermidis**, Rodilla
$\text{BERTDeCS}_{\text{CL+PT+FT}}$ (F1: 0.667)	**Teicoplanina**, **Humanos**, **Prótesis de la Rodilla**, Artroplastia de Reemplazo de Rodilla, Antibacterianos, **Masculino**, **Staphylococcus epidermidis**, **Infecciones Relacionadas con Prótesis**, Rodilla, **Farmacocinética**

7.1 Prompt Template for Qwen-based DeCS Annotation

The following prompt was used for the Qwen2.5-7B-Instruct experiments:

Eres un indexador biomédico especializado en DeCS/MeSH.

Tu tarea es asignar descriptores DeCS relevantes a un artículo biomédico en español, usando solamente el título y el resumen.

Reglas:

1. Devuelve solamente nombres de descriptores DeCS en español.
2. No inventes etiquetas libres, frases demasiado específicas ni explicaciones.
3. Prioriza los temas principales del artículo, incluyendo enfermedades, organismos, sustancias químicas, métodos, tipos de estudio, poblaciones y procedimientos clínicos.
4. Devuelve como máximo 20 descriptores.
5. La salida debe ser únicamente JSON válido.
6. No incluyas texto fuera del JSON.

Artículo:

Título:

{title}

Resumen:

{abstract}

Devuelve JSON exactamente en este formato:

```
{
  "descriptors": [
    "descriptor 1",
    "descriptor 2"
  ]
}
```
